# Sustained conduction of vasomotor responses in rat mesenteric arteries in a two‐compartment in vitro set‐up

**DOI:** 10.1111/apha.13099

**Published:** 2018-06-17

**Authors:** T. Palao, A. van Weert, A. de Leeuw, J. de Vos, E. N. T. P. Bakker, E. van Bavel

**Affiliations:** ^1^ Department of Biomedical Engineering and Physics Academic Medical Center Amsterdam the Netherlands

**Keywords:** conducted responses, resistance arteries, vasoconstriction, vasodilation

## Abstract

**Aim:**

Conduction of vasomotor responses may contribute to long‐term regulation of resistance artery function and structure. Most previous studies have addressed conduction of vasoactivity only during very brief stimulations. We developed a novel set‐up that allows the local pharmacological stimulation of arteries in vitro for extended periods of time and studied the conduction of vasomotor responses in rat mesenteric arteries under those conditions.

**Methods:**

The new in vitro set‐up was based on the pressure myograph. The superfusion chamber was divided halfway along the vessel into two compartments, allowing an independent superfusion of the arterial segment in each compartment. Local and remote cumulative concentration‐response curves were obtained for a range of vasoactive agents. Additional experiments were performed with the gap junction inhibitor 18β‐glycyrrhetinic acid and in absence of the endothelium.

**Results:**

Phenylephrine‐induced constriction and acetylcholine‐induced dilation were conducted over a measured distance up to 2.84 mm, and this conduction was maintained for 5 minutes. Conduction of acetylcholine‐induced dilation was inhibited by 18β‐glycyrrhetinic acid, and conduction of phenylephrine‐induced constriction was abolished in absence of the endothelium. Constriction in response to high K^+^ was not conducted. Absence of remote stimulation dampened the local response to phenylephrine.

**Conclusion:**

This study demonstrates maintained conduction of vasoactive responses to physiological agonists in rat mesenteric small arteries likely via gap junctions and endothelial cells, providing a possible mechanism for the sustained functional and structural control of arterial networks.

## 1 INTRODUCTION

Resistance arteries and arterioles are organized in vascular networks. These vascular networks are dynamic, and spatial coordination of arteriolar tone is essential for regulation of flow in response to alterations in metabolic need.^1^ Thus, impaired coordination under pathological conditions may contribute to insufficient local perfusion. Conducted responses may form the base for spatial coordination of local vasomotor responses.^2^ Conduction of both vasoconstriction and vasodilation in vascular networks has been described,^3^ and the mechanisms through which these occur are subject of ongoing studies. It has been shown that changes in membrane potential (hyper‐ or depolarization) resulting from local stimuli can propagate intercellularly mainly along the endothelial cells, but also along the smooth muscle cell layers of arteries.^4^ Gap junctions allow the cell‐cell coupling of membrane potential, providing a possible mechanism for facilitating such propagation. Alternatively, transport of small solutes such as calcium ions through gap junctions may contribute to conducted vasomotor responses^5^, ^6^, ^7^, ^8^ Conduction is believed to be independent of vascular innervation or mechanical signalling.^9^, ^10^ These conducted responses are able to spread downstream and upstream over vascular nodes and branches.^11^

Studies on conduction of vasomotor responses have been carried out both in vivo and in vitro, and in different vascular beds. Although in many studies the local stimulus is produced by the addition of an agonist, other studies also used local electrical stimulations.^1^, ^10^ In the first case, drugs, such as phenylephrine or acetylcholine, are delivered by placing the tip of a micropipette next to an artery, assuring that the agonist is kept away from the remote site by a superfusion flow in the opposite direction. In all cases, stimulations were localized and brief, consisting of short pulses of up to few seconds, while remote responses were recorded at variable distances from the stimulated site (normally 500‐2000 μm).^12^

These studies have been essential to characterize and understand the mechanisms underlying conducted vasomotor responses. Yet almost all work has been limited to the conducted responses to a brief stimulation, typically <1 second. However, coordination of tone is also required at a time scale in the order of minutes to hours. Moreover, modelling studies have shown the importance of conducted responses in structural adaptations in vascular networks^13^, ^14^ To the best of our knowledge, only few earlier studies addressed longer lasting stimulation, demonstrating maintenance of conducted vasodilator responses up to two minutes.^15^, ^16^ The conduction of vasomotor responses during longer periods has not been studied so far. Thus, in our study, we present a novel set‐up that allows the local pharmacological stimulation for extended periods of time in small arteries in vitro and studied the conduction of vasomotor responses in mesenteric arteries from rats along a concentration‐response curve after 5 minutes of stimulation per concentration.

## 2 RESULTS

### 2.1 Leakage quantification

**Figure 1:**
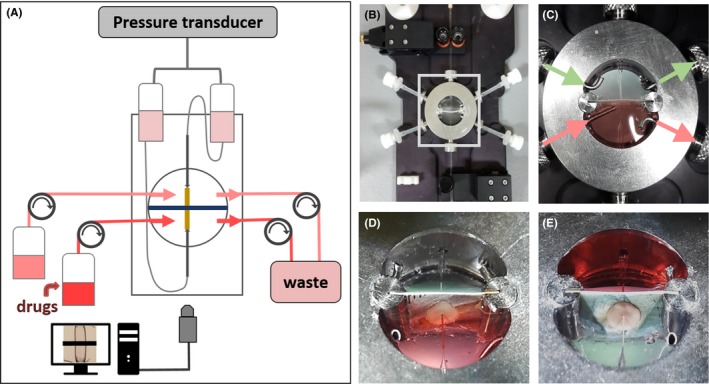
Description of the set‐up. A, Schematic drawing of the set‐up and the perfusion system. B, C, Detail of the chamber and the superfusion inlets and outlets. D, E, Details of the septum that divides the chamber, showing absence of leakage of the Ponceau‐red solution through the septum

To quantify potential leakage of drugs to the unstimulated, remote part of the vessel, the concentration of fluorescein was measured in both compartments (see Figure 1 described in the methods for the set‐up description). Only 0.023 ± 0.008% (n = 10) of the fluorescence intensity was detected in the samples of medium coming from the remote compartment, implying a minimal leakage of drug and thus assuring the local nature of stimulation of the arterial segment.

### 2.2 Viability

**Figure 2:**
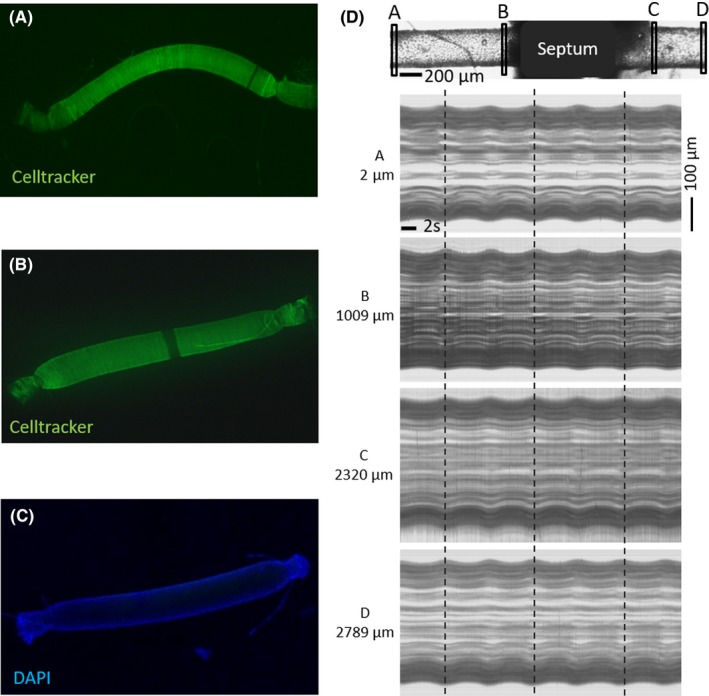
Vessel viability. A, Cannulated artery incubated with CellTracker (green) showing no damage in the septum area. B, Cannulated artery with deliberate damage in the centre as control. C, Negative control without CellTracker incubation. D, Analysis of the synchronized vasomotion along the artery. The top image shows the 4 points where the analysis was performed. The 4 lower images show the line scans of the vessel contour over the time. Dashed lines are placed at moments of peak dilation during the vasomotion, and these moments occur simultaneously on both sides of the septum over a total distance of 2.8 mm

Vessel viability was tested before every experiment with U46619 and bradykinin, and non‐responsive vessels were discarded. Extra tests to ensure whether the septum could affect the viability of the artery and the cellular communication along the vessel were performed. Images of the vessels mounted on the set‐up did not show any damage or cellular death in the septum area of the vessel when stained with a living cell fluorescent probe (“CellTracker”; Figure 2A‐C). Cell‐to‐cell communication was also studied by inducing vasomotion in cannulated arteries in the set‐up. Once the vasomotion was induced, a synchronization of the oscillations in both arterial segments was observed (Figure 2D). Based on frame rate and length of the vessel, wave speed of vasomotion was estimated to be at least 2 cm/s. The supplementary video provides an example (Video S1).

### 2.3 Conduction of vasoconstriction

**Figure 3:**
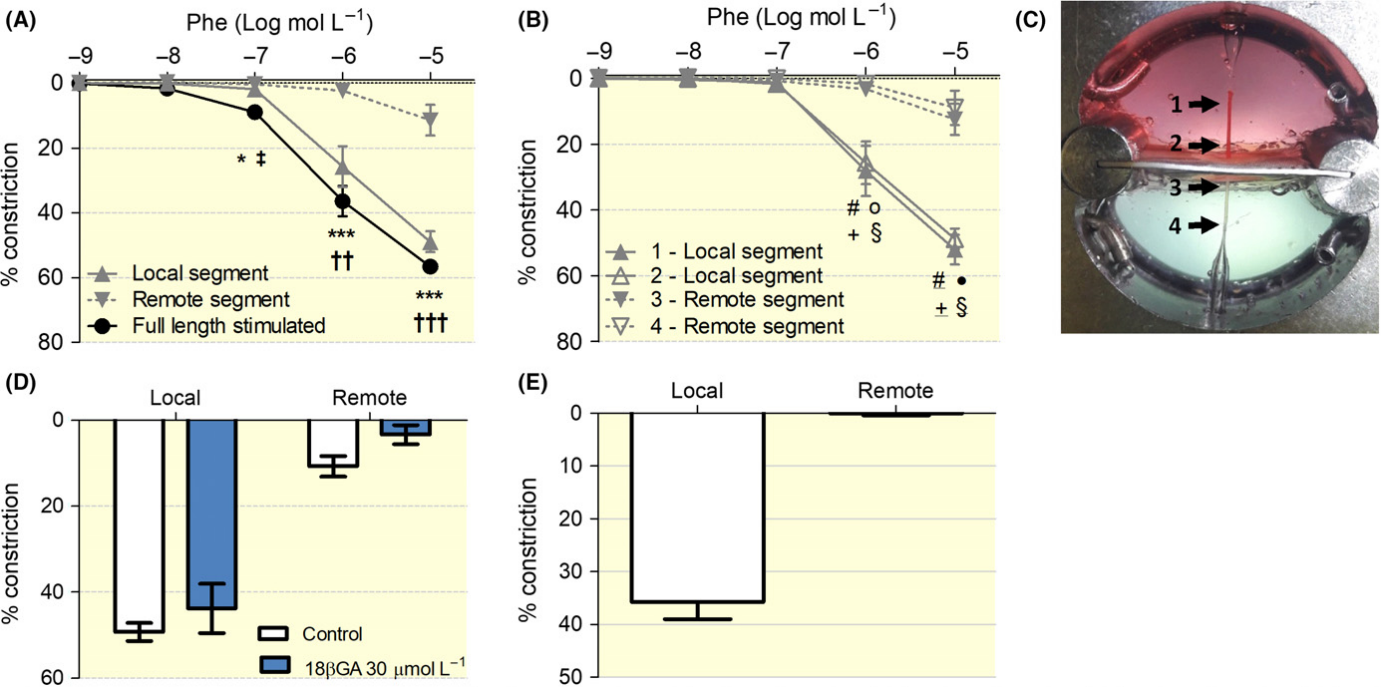
Conduction of local phenylephrine‐induced vasoconstriction. A, Concentration‐response curves to phenylephrine (phe) for both fully (black) and locally (grey) stimulated vessels. B, Responses at the four locations to local stimulation at locations 1 and 2 (n = 7 full length stimulated; n = 9 locally stimulated). C, Image of the set‐up indicating the different measurement points along the arteries with arrows. D, Local and remote responses of vessels locally stimulated with 10^−5^ mol L^−1^ phe with and without the presence of the gap junction inhibitor 18β‐glycyrrhetinic acid (n = 5). E, Local and remote responses of endothelium removed vessels locally stimulated with 10^−5^ mol L^−1^ phe (n = 4). *P < .05 remote segment vs full length; **P < .01 remote segment vs full length ***P < .001 remote segment vs full length; †*P* < .05 remote segment vs local segment; ††*P* < .01 remote segment vs local segment; †††*P* < .001 remote segment vs local segment; ‡*P* < .05 local segment vs full length. #*P* < .05 4‐remote segment vs 1‐local segment; # *P* < .01 4‐remote segment vs 1‐local segment; o *P* < .05 4‐remote segment vs 2‐local segment; ● *P *< .01 4‐remote segment vs 2‐local segment; +*P *< .05 3‐remote segment vs 1‐local segment; ±*P* < .01 3‐remote segment vs 1‐local segment; §*P* < .05 3‐remote segment vs 2‐local segment; § *P* < .01 3‐remote segment vs 2‐local segment

Conduction of local vasoconstriction was studied with the α_1_‐adrenergic receptor agonist phenylephrine and the depolarizing KCl solution. In the concentration‐response curve to phenylephrine, a constriction of the remote part of the vessel was observed at only the higher drug concentrations (Figure 3A). Vascular responses were measured at four points along the arteries, as schematically indicated in Figure 3C. No significant differences in constriction were found between the two local sites (close to the septum and close to the pipette; Figure 3B). In the remote segment, constriction decayed with distance from the septum. Although, when tested as part of the ANOVA, the difference was not significant between the two remote sites, located at respectively 1.62 ± 0.57 and 2.84 ± 1.00 mm from the septum, responses were smaller at the more remote location in 8 of 9 vessels, with a missing observation in the 9th vessel. An attempt to fit a mono‐exponential decay over the vessel length to these conducted responses provides an estimated length constant of 1.41 mm. Yet, as shown in Figure S1, the decay is not adequately described by such a mono‐exponential function. A graph and a video illustrating the development over time of the conduction of the local stimulation with 10^−6^ mol L^−1^ phenylephrine can be found in Figure S2 and Video S2 respectively. The conduction of the responses at 1.55 ± 0.29 mm from the septum showed a tendency to become attenuated in the presence of the gap junction inhibitor 18b‐glycyrrhetinic acid (18βGA; Figure 3D) and was not observed in arteries lacking of endothelial layer (Figure 3E).

A lack of remote stimulation may dampen the local response. Indeed, the response of the locally stimulated segment (grey) of the artery was smaller as compared to the full length stimulated vessel (black), assessed as an average of the measurements made along all the stimulated vessel. This difference was statistically significant at intermediate doses (Figure 3A).

**Figure 4:**
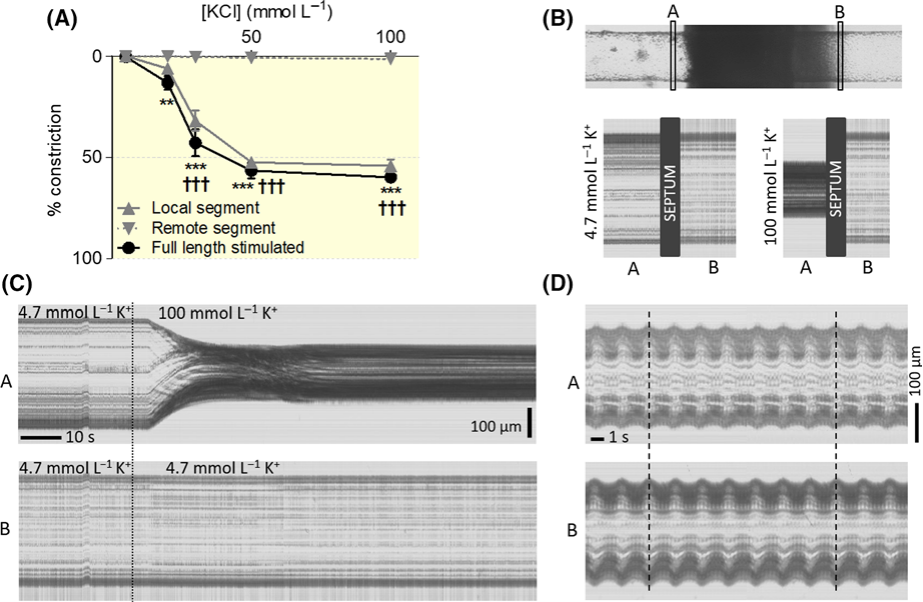
Conduction of local K^+^‐induced vasoconstriction. A, Concentration‐response curve to K^+^ in both fully and locally stimulated vessels (n = 4 full length stimulated; n = 5 locally stimulated). B, Upper part: Image of one of the vessels used to study transient responses to K^+^. A B, indicate the positions used in the line scan in (C) and (D). Lower part: Images from the slices indicated above at the start and at the end of the experiment, showing the constriction of the local but not the remote site to 100 mmol L^−1^ K^+^.(C) Images showing the line scans of the vessel contour at A and B over the time during local stimulation with high K^+^ concentration. (D) Images showing the synchronized vasomotion in both sites in this particular vessel. **P < .01 remote segment vs full length ***P < .001 remote segment vs full length; †††*P* < .001 remote segment vs local segment

In the case of the K^+^, sustained conduction of the constriction was not observed at any concentration (Figure 4A) at both remote locations situated at 1.39 ± 0.62 mm and 2.80 ± 1.25 mm from the septum. Here, we also observed a tendency to lower constriction in the locally stimulated segment as compared to arteries that were stimulated over the full length. Transient responses to 100 mmol L^−1^ K^+^ were studied by observation of the whole vessel length at 2.5× magnification rather than by repositioning the microscope to various locations at 10×, as was done for the sustained responses. While the arteries showed synchronized vasomotion, demonstrating intercellular communication (Figure 4D, Video S4), local stimulation with 100 mmol L^−1^ of K^+^ did not produce any transient response at the remote site (Figure 4B,C, Video S3) in 3 tested vessels.

### 2.4 Conduction of vasodilation

**Figure 5:**
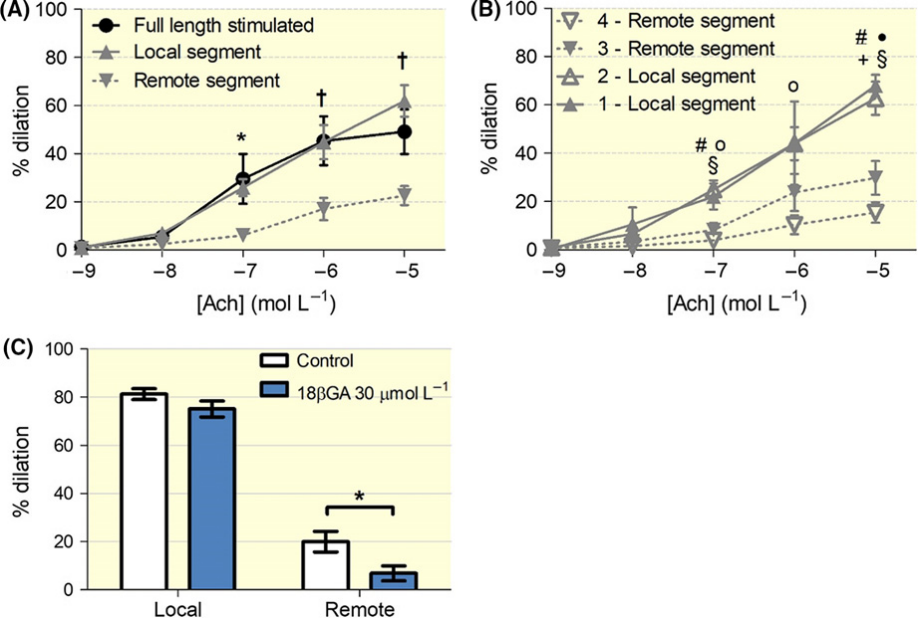
Conduction of vasodilation. A, concentration‐response curves to acetylcholine (Ach) for both fully (black) and locally stimulated vessels (grey). (n = 5 full length stimulated; n = 7 locally stimulated). B, Responses to local acetylcholine at the four locations. C, Local and remote responses of vessels locally stimulated with 10^−5^ mol L^−1^ Ach with and without the presence of the gap junction inhibitor 18β‐glycyrrhetinic acid (n = 5). For panel (A) and (B): *P < .05 remote segment vs full length; †*P* < .05 remote segment vs local segment. #*P* < .05 4‐remote segment vs 1‐local segment; # *P* < .01 4‐remote segment vs 1‐local segment; o *P* < .05 4‐remote segment vs 2‐local segment; ● *P *< .01 4‐remote segment vs 2‐local segment; +*P *< .05 3‐remote segment vs 1‐local segment; §*P* < .05 3‐remote segment vs 2‐local segment. For panel (C): *P < 0.05 control vs 18βGA

To study conduction of vasodilatory responses, arteries were locally stimulated with increasing doses of acetylcholine after 10^−6^ mol L^−1^ phenylephrine preconstriction in both the local and remote segments. A clear conduction of the response was observed in the remote segment (Figure 5A). Figure 5B provides data for the two remote spots, located at 1.37 ± 0.52 mm and 2.69 ± 1.02 mm from the septum. The decay over distance could be described a mono‐exponential function and the length constant calculated for the conduction of the response to acetylcholine at concentrations of 10^−6^ mol L^−1^ and 10^−5^ mol L^−1^ were, respectively, 2.00 and 1.87 mm (Figure S1). A graph and a video illustrating the development of the conduction of the local stimulation with 10^−6^ mol L^−1^ acetylcholine can be found in Figure S3 and Video S5 respectively. The conduction of vasodilation in response to local stimulation with acetylcholine at 1.55 ± 0.29 mm from the stimulated site was significantly decreased when the arteries were incubated with 18βGA (Figure 5C). Endothelium‐denuded vessels did not respond to acetylcholine (not shown). Unlike found for phenylephrine, the absence of remote stimulation did not significantly impair the local vasodilator response to acetylcholine.

## 3 DISCUSSION

In this study, we developed a new set‐up that allows local stimulation of arteries in vitro for extended periods of time. We used this set‐up to study the sustained conduction of vasomotor responses in mesenteric arteries from rats and recorded local and remote concentration‐response curves. We observed a conduction of vasoconstriction and vasodilation in response to local stimulation with, respectively, phenylephrine and acetylcholine that decayed along the vessel, while conduction of response to K^+^ was not present.

Conduction of vasomotor responses in resistance artery networks has been widely described.^17^, ^18^, ^19^, ^20^ These responses are believed to play an important role in coordination of arterial tone in response to stimuli such as metabolic demand during exercise, thus regulating blood flow.^1^ Typical experiments on conduction consist of a very localized application of drugs with a small micropipette in both in vivo and in vitro preparations.^12^, ^21^ The experimental protocols used so far included very short local stimulation and observation of remote responses, mimicking a short‐lasting local stimulus that is received in the distal circulation and transmitted upstream to the arterial networks. However, there are good arguments to believe that tone in vascular networks also needs to be controlled at longer time scales. Notably, myogenic tone and sympathetic drive are among the factors that set the baseline level of tone.^22^ A highly heterogeneous baseline tone would not only cause a disturbed distribution of flow in networks, but also a strongly increased peripheral resistance. This is the case because, according to the Poiseuille relation, local resistance is inversely proportional to the fourth power of the diameter and small sites of deep constriction would therefore strongly add to resistance. Conduction of baseline tone may thus act as a spatial low‐pass filter, smoothing out tone irregularities over a length of a few mm. Similarly, the vasodilator influences of among others metabolically active tissue have a tonic component too. To be effective, such continuous vasodilation needs to be conducted to establish dilation over sufficient distance. Finally, sustained tone influences vascular remodelling, with deep tone being a drive for inward remodelling.^23^, ^24^ Previous modelling studies have shown that conducted responses, as important tone regulators, may play a role in vascular structural adaptations.^13^, ^25^ Speculatively, conduction of tone could thus result in a smoothing out of irregularities in vascular structure and calibre. Theoretical work has suggested a role for such conducted remodelling in arteriolar network stability.^14^

Despite this anticipated role in vascular regulation, little is known about coordination of tone for longer periods of time. To the best of our knowledge, there are few studies that performed longer stimulations up to 2 minutes.^15^, ^16^ We performed concentration‐response stimulations, with several concentration steps of 5 minutes each, thus stimulating for much longer total periods of time. With this approach, we aimed to observe conducted responses in extended periods of stimulation time under well‐defined concentrations. This study provides a step towards exploring the role of conduction in long‐term vascular adaptations. It remains to be established whether continuous changes in tone remain conducted over days, thereby providing a base for possible “conducted remodelling.”

### 3.1 Differences between agonists, between beds

Conduction of vasoconstriction in response to brief application of phenylephrine was previously observed at high doses (10^−4^ mol L^−1^) in hamster cheek pouch in vivo preparations.^26^ In those experiments, the length constant for phenylephrine was calculated to be 1.9 mm. Our remote sites (1.62 and 2.84 mm) covered this distance and indeed a partial and conduction was found that decayed with distance. However, we could not fit our data for phenylephrine by a mono‐exponential decay. It remains to be established why this was the case.

Gap junction inhibition with 18βGA produced a tendency to a decrease in the conduction of the responses, pointing to a gap junction‐mediated communication. It should be noted that although 18βGA has been widely used as gap junction inhibitor,^27^ this compound, as many of the gap junction blockers, may have non‐specific effects^28^ and clearly, future work should include more robust testing of their role, also by including membrane potential recordings.

Local stimulation of endothelium‐denuded arteries did not produce remote phenylephrine responses, suggesting a role for the endothelium in the sustained conduction of phenylephrine‐induced vasoconstriction. So far, literature suggests that conduction of phenylephrine occurs via the smooth muscle cell layer.^5^ Our endothelium‐denuded vessels had normal local responses, arguing against damage of the smooth muscle cells. However, we cannot rule out that the intimal rubbing may have altered the optimal state of the vessel, influencing the cell‐to‐cell communication. Clues towards a role for the endothelium in conducted phenylephrine responses include the transient local and conducted depolarizations in both smooth muscle and endothelial layers in response to phenylephrine.^29^ Moreover, induced changes by phenylephrine in smooth muscle [Ca^2+^]_i_ may influence the level of Ca^2+^ in adjacent endothelial cells.^30^

Our results on maintained conduction of acetylcholine‐induced vasodilation are in accordance with observations following short stimulation in several vascular beds,^21^, ^31^, ^32^ and in fact, acetylcholine is one of the most frequently used vasodilators to study conducted vasodilation. In previous studies using rat mesenteric arteries under transient stimulation, conduction of the acetylcholine response was shown to reach up to 2000 μm.^33^, ^34^ We now show that this response is sustained that the sustained response is concentration‐dependent and well fitted by an exponential decay in accordance with the above studies. The suppression of this sustained response by 18βGA suggests involvement of gap junctions, again with the note that this blocker may have unspecific effects.

In our hands, local stimulation with increasing concentrations of K^+^ showed no conduction. Additionally, no initial transient changes were observed when recording at up to 7 frames per second and in response to direct exposure to high concentrations of K^+^ instead of cumulative concentration‐response curves. Based on the viability tests performed and the observation of conduction of other stimuli in our set‐up, we do not believe that the vessels might have been damaged, disrupting the cell‐cell communication. Conduction of responses to local K^+^ has been observed in hamster cheek pouch and rat kidney arterioles,^35^, ^36^ as well as in mesenteric arterioles.^37^ In this latter case, terminal arterioles were used (fifth order branch). In our case, due to the limitations of the in vitro set‐up, second‐ and third‐order branch arteries were used, as the vessels need to have sufficiently length without side branches to allow cannulation, progressing through the septum and monitoring local versus remote responses. Conduction of vasomotor responses has been shown to vary between vascular beds and also between agonists used.^10^, ^26^ The larger diameter and different architecture of the vessels we used may alter the membrane properties of those arteries and thus may explain the absence of conduction of K^+^ stimulus in our hands. However, it was not the scope of the present work to study differences in conducted responses among vascular beds and branching orders.

For both phenylephrine and acetylcholine cases, although we stimulated half of the vessel rather than only a very local site, the similarity of length constants to previous studies suggests that stimulation over a longer vessel length does not induce a remote response that reaches further.

### 3.2 Two faces of conduction: impairment of local effects

Interestingly, we detected lower responses to phenylephrine in the locally stimulated segment of the artery as compared to the full length stimulated vessel, especially at intermediate doses. This suggests that the unstimulated part of the vessel “talks back” and reduces constriction in the stimulated part. Thus, communication may act in two ways: activation is conducted, but remote lack of stimulation also suppresses the local vasoconstriction, reminiscent of a “current sink.” Such behaviour would indeed be expected if the vessel could be represented as a network of linear time‐invariant membrane and gap‐junctional conductances. Future modelling work could help understanding whether such behaviour can also be expected in a complex network of endothelial and smooth muscle cells with non‐linear and time‐varying conductances. An analogous reduced local vasodilator effect in the absence of remote stimulation was not observed when the vessels were stimulated with acetylcholine. Speculatively, these data may point towards a modus operandi of resistance vessels where baseline tone is integrated and smoothed over distance while local dilator responses in the distal vessels can develop despite the presence of unstimulated larger vessels and can be conducted towards these larger vessels. A recent review addresses both the mechanisms and the (patho)physiological role of conducted responses^38^ and clearly indicates the need for computational models of conduction in segments and networks that address these issues.

### 3.3 Limitations

It is obviously fundamental that the drug application remains local, to ensure that the remote effects are due to signal conduction. In the previous micropipette studies, this was assured by keeping both intra‐ and extraluminal flow directed away from the measurement site. We established negligible leakage via the septum, using a flexible latex membrane with a small hole to allow passing the artery. We ensured that intraluminal and superfusion flows always were directed towards the local site. The level of spill over that was found (average of 0.023%) was minimal and cannot account for the observed remote responses. While we needed a tightly closing membrane for this, we demonstrated the lack of cell death due to a potential mechanical damage of the vessel by the septum present in our set‐up.

The present set‐up allowed the imaging of only a part of the artery at a time, while direct observation of the full length of vessels at a low magnification implied a loss in image resolution (3 μm), which is too limited to be used for the concentration‐response studies. An alternative imaging strategy that allows the continuous high‐resolution readout of the full arterial segment is needed for the rigorous testing of dynamic responses and decay of initial and sustained responses over the length of the vessel.

A limitation of our study is the size of the vessels. These need to have a diameter large enough to be cannulated and a length sufficient to cross the septum, making some vascular beds and small arterioles not suitable for this approach. Perivascular innervation might play a role in the conduction of signal in vessels of the current size. Future studies should investigate this role. Fluorescence‐based imaging of calcium and membrane potential is among the tools that could help elucidating the mechanisms of sustained conduction, but are unfortunately not possible in our current set‐up that requires long working distance objectives, temperature control by an incubator rather than heated chamber, and that misses imaging options with high sensitivity and high frame rate.^37^ Very brief stimulations mimicking previous work could not be performed since a few seconds were needed to replace the medium in the chamber. Lastly, we studied an isolated segment, not connected to a vascular network. While this is a general limitation of cannulated vessel studies, the current study raises the question to what extent the absence of connected vessels influences the in vitro responsiveness of these vessels.

In conclusion, we present a novel set‐up to study conduction of sustained local stimulations. With this approach, differential conduction of vasoconstriction, but clear conduction of vasodilation was observed in rat mesenteric arteries. We showed that conducted responses occur across a full concentration‐response relationship, persist at least for several minutes and are likely mediated by gap junctions and the endothelium. In addition, we showed that absence of remote stimulation dampens the local response to vasoconstrictors.

## 4 MATERIAL AND METHODS

### 4.1 Animals

All experiments were approved by the Committee for Animal Experiments of the Academic Medical Center, Amsterdam. Male Wistar rats (461.9 ± 10.7 g) were obtained from Charles River, the Netherlands, and used at 16.2 ± 0.5 weeks of age. All the animals were housed in the animal care facility of the Academic Medical Center with 12:12 hour light‐dark cycle at RT and access to food and water ad libitum.

### 4.2 Tissue collection

Animals were anesthetized with isoflurane (5%), followed by decapitation. The intestines were removed and placed in cold MOPS‐buffered physiological salt solution (in mmol L^−1^: 145.0 NaCl, 4.7 KCl, 1.17 MgSO_4_, 1.2 NaH_2_PO_4_, 2 CaCl_2_, 5.0 glucose and 2.0 pyruvate; pH 7.35). Second‐ and third‐order branches of the mesenteric artery (diameter 397 ± 15 μm; total length 8.00 ± 0.39 mm; length local segment (see below for definition) 3.74 ± 0.36 mm; length remote segment 3.21 ± 0.19 mm) were dissected using a stereomicroscope. The vessels were cleaned from adipose tissue and blood and kept in cold Ca^2+^ free MOPS solution until cannulation.

### 4.3 Conducted vasomotor responses set‐up

To study conducted responses, we designed a new in vitro set‐up consisting of a modification of the pressure myograph (Figure 1A). In pressure myography, arteries are cannulated between glass micropipettes in a chamber with physiological medium at 37°C, and intraluminal pressure and flow are controlled. The bottom of the chamber was made of quartz glass to allow visualization of the vessel via an objective connected to a camera. In this modified set‐up, the superfusion chamber was divided into two compartments by a septum halfway along the vessel (Figure 1B). The superfusion system in each compartment was independent, thus allowing a vasoactive stimulus to be applied locally in one of the compartments (“local”) while the other part of the artery (“remote”) remained unstimulated (Figure 1C). The centre of the septum was covered by a very thin latex layer with a pinhole through which the vessel crosses (Figure 1D,E). To mount the vessel in this system, we first cannulated one end. The free end of the vessel was then advanced towards the septum by moving the micropipette. Elasticity of the thin latex layer allowed opening the pinhole with a forceps while passing the artery during mounting, by further advancing the micropipette and sideward steering by a second forceps. Once the vessel was in place, the latex membrane was released, which then very gently sealed the gap without causing any deformation of the vessel, minimizing the risk of damage. Finally, the other end of the vessel was cannulated. Care was taken never to move the vessel through the pinhole after this initial positioning.

The fluid level was always kept higher in the remote compartment, such that any remaining fluid leakage occurs towards the local compartment, keeping the remote compartment clear of drugs. Drugs were added by replacing the inflow of the superfusion solution. The inlet tube in the stimulated compartment was directly aimed at the artery, to allow more rapid concentration changes.

The vessel was imaged one location at a time, moving the objective along the artery. The diameter of the vessels was assessed by straightforward edge detection of the outer contour, with subsequent constrained edge detection of the inner contour. This was carried out on‐line over lengths of typically 100 μm at particular locations. The measurements were made with a 10× NA 0.25 dry objective in an inverted setting, providing a resolution of 1.1 μm. Pixel resolution was 0.65 μm/pixel. The field of view along the vessel length was 825 μm. In additional experiments aimed at simultaneous observation of both locations and testing transient remote responses and synchronization of vasomotion, we used a 2.5× NA 0.08 objective, covering 3.3 mm of vessel length at a resolution of 3 μm, or a upright dissection microscope, covering 8.4 mm of vessel at a resolution of 6 μm.

### 4.4 Leakage quantification

To quantify the amount of drug that could pass through the septum to the remote compartment, arteries were mounted in the set‐up and pressurized. The locally stimulated part of the vessel was superfused with Leibovitz′s L‐15 medium (Gibco, Life Technologies) containing 0.1% dextran, fluorescein‐labelled (500 kD, Ex. 494 nm/Em. 521), (Molecular Probes‐Life Technologies) during 5 minutes at high speed rate (80 rpm), simulating the stimulation steps in the experimental procedure described later. After that time, samples of medium from both local and remote compartments were taken and fluorescence intensity was measured with spectrophotometry.

### 4.5 Viability

Viability of the arteries was tested before every experiment. Smooth muscle contractile function was tested with the thromboxane A2 analogue, U‐46619 (Sigma‐Aldrich). Endothelium‐dependant vasodilator responsiveness was tested with bradykinin (Sigma‐Aldrich). Non‐responsive vessels were discarded.

To verify the absence of vascular damage due to the passage through the septum, some arteries were incubated with the living cell fluorescent probe CellTracker™ Green CMFDA (Thermo Fisher), 1 μmol L^−1^ for 30 minutes at 37°C. Afterwards, the vessels were fixed with 3.7% paraformaldehyde and mounted in a microscopy glass with VectaShield mounting media (Vector Laboratories) with DAPI staining to visualize nuclei. Arteries were visualized in a fluorescence microscope.

To check for cell viability and communication, we also studied the induction of coordinated smooth muscle oscillations (vasomotion) in a separate group of cannulated arteries. The vessels were stimulated with 10^−6^ mol L^−1^ noradrenaline, which induced constriction and vasomotion. Vasomotion in both parts of the artery was visualized simultaneously, with a 2.5× NA 0.08 objective, covering 3.3 mm of vessel length at a resolution of 3 μm, and synchronization of vasomotion was considered to reflect intact communication between the both parts.

Videos of the synchronization were recorded as image sequence (7 fps) with a 2.5× objective. Analysis of the videos was performed with Image J, by performing a reslicing of the image sequence, obtaining line scans of the vessel contour at several spots along the artery over time.

### 4.6 Local drug application

Mesenteric arteries were cannulated in the set‐up and pressurized to 60 mm Hg. After viability was tested, arteries were locally stimulated by replacing the superfusion medium of the stimulated compartment with medium containing increasing concentrations of the drugs to test. To study conducted responses after extended periods of stimulation, a 5 minutes stimulation period was applied per concentration. After the 5 minutes, at each concentration, the inner and outer diameters were measured at 4 different locations along the vessel: close to the cannula tip and as close as possible to the septum in both the local and remote compartment. Responses in these arteries were compared to vessels that were stimulated in both compartments.

In all experiments, except in the case of stimulation with potassium, arteries were maintained in Leibovitz′s L‐15 medium. Phenylephrine (Sigma) was applied in increasing concentrations. To study conduction of vasodilation, arteries were first constricted with phenylephrine 10^−6^ mol L^−1^ in both compartments. Once a stable constriction was achieved, the artery was additionally locally stimulated with increasing concentrations of acetylcholine (Sigma).

For the high K^+^ experiments, the arteries were superfused with MOPS buffer. To stimulate the arteries, MOPS buffers with increasing concentrations of KCl (5, 20, 30, 50 and 100 mmol L^−1^) were used, prepared with equimolar replacement of NaCl by KCl. When studying transient responses to high KCl concentration, arteries were superfused with normal MOPS buffer and then locally stimulated with 100 mmol L^−1^ KCl MOPS buffer. Images were taken at 7 fps and analysed as described above for vasomotion.

For the experiments with the gap junction inhibitor 18β‐glycyrrhetinic acid (18βGA) (Sigma), arteries were locally stimulated with phenylephrine 10^−5^ mol L^−1^ for 5 minutes, and responses were recorded at the end of that period in both local and remote compartments. Next, the arteries were preconstricted with phenylephrine 10^−6^ mol L^−1^ in both compartments and locally stimulated with acetylcholine 10^−5^ mol L^−1^ for 5 minutes, after which responses were recorded in both local and remote compartments, followed by wash‐out. Arteries were then incubated with 3 × 10^−5^ mol L^−1^ 18βGA in both compartments for 30 minutes, and stimulations with local phenylephrine and acetylcholine were again performed in the continuous presence of the inhibitor.

To remove the endothelium, a rat whisker was passed through the lumen of the arteries prior to cannulation. Endothelial removal was tested with 10^−6^ mol L^−1^ acetylcholine, and arteries that still showed dilation were discarded. Denuded arteries were locally stimulated with phenylephrine 10^−5^ mol L^−1^ for 5 minutes, and at the end of that period, responses were recorded in both local and remote compartments.

### 4.7 Statistics

Data are expressed as mean ± SEM. Data were analysed using Student's *t* test or one‐way ANOVA followed by Bonferroni post hoc test or two‐way ANOVA with repeated measurements, with GraphPad Prism 5 software. A *P*‐value of <.05 was considered statistically significant. We attempted to determine a length constant for conduction, by fitting the mean conducted responses by a mono‐exponential function of the form *y* = y_0_∙exp(−*x*/λ), with *y* the remote effect, y_0_ the local effect, *x* the distance from the septum in the remote part of the vessel, and λ the length constant.

## CONFLICT OF INTEREST

None of the authors have any conflict of interest to declare.
