# Supplementary figures and images for: Sustained conduction of vasomotor responses in rat mesenteric arteries in a two‐compartment in vitro set‐up
Source: Acta Physiol (Oxf). 2018 Jun 17;224(3):e13099. doi: 10.1111/apha.13099 (PMC6221078; doi:10.1111/apha.13099)

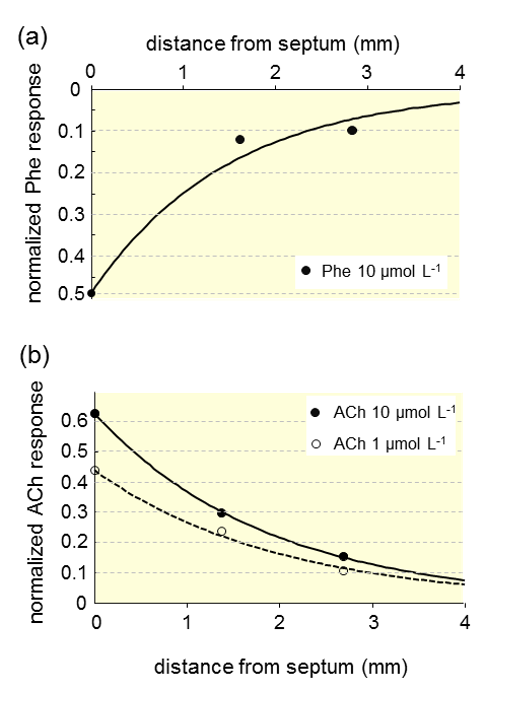

Supplement: Supplementary file 2 [file APHA-224-na-s002.tif]

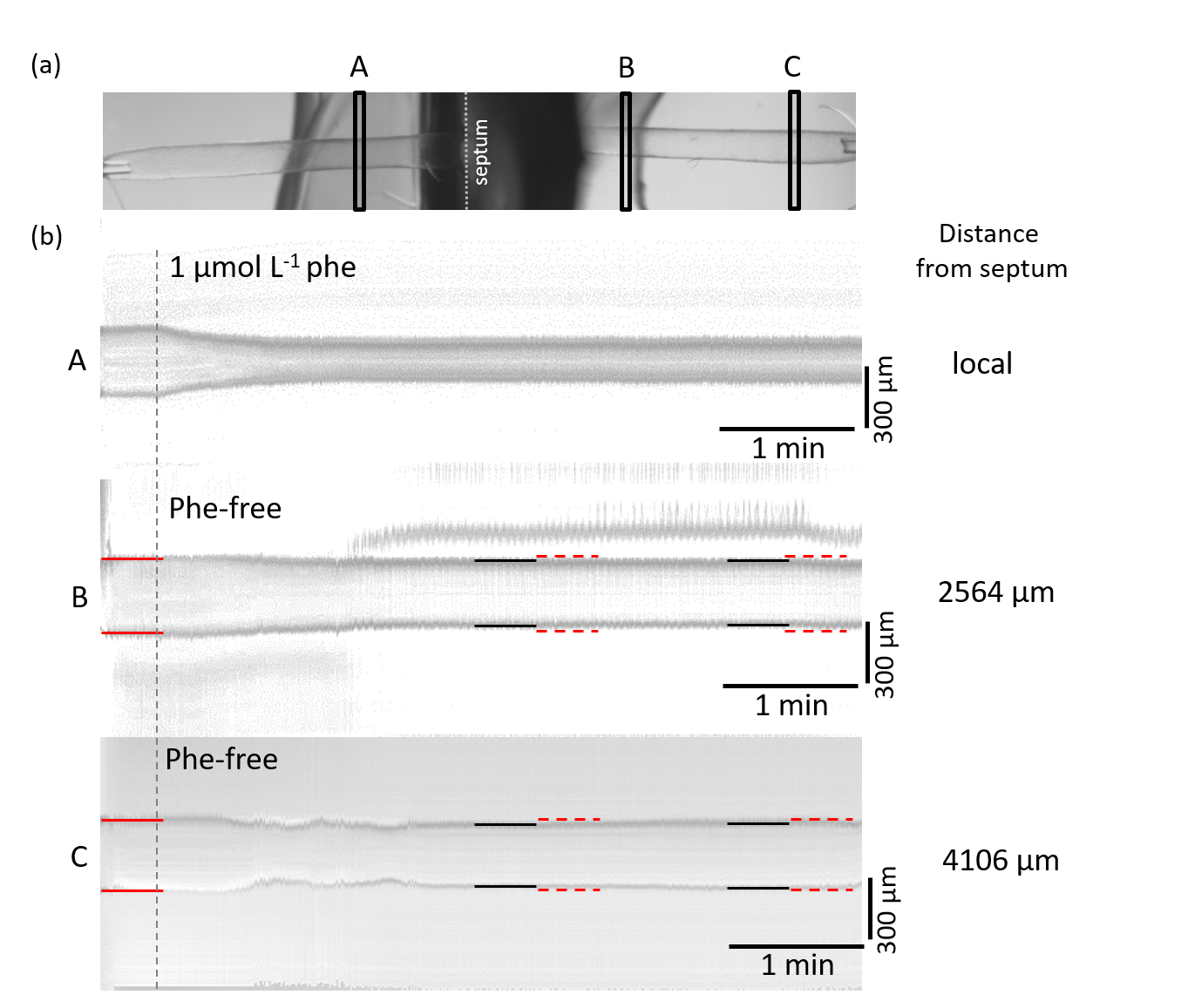

Supplement: Supplementary file 3 [file APHA-224-na-s003.tif]

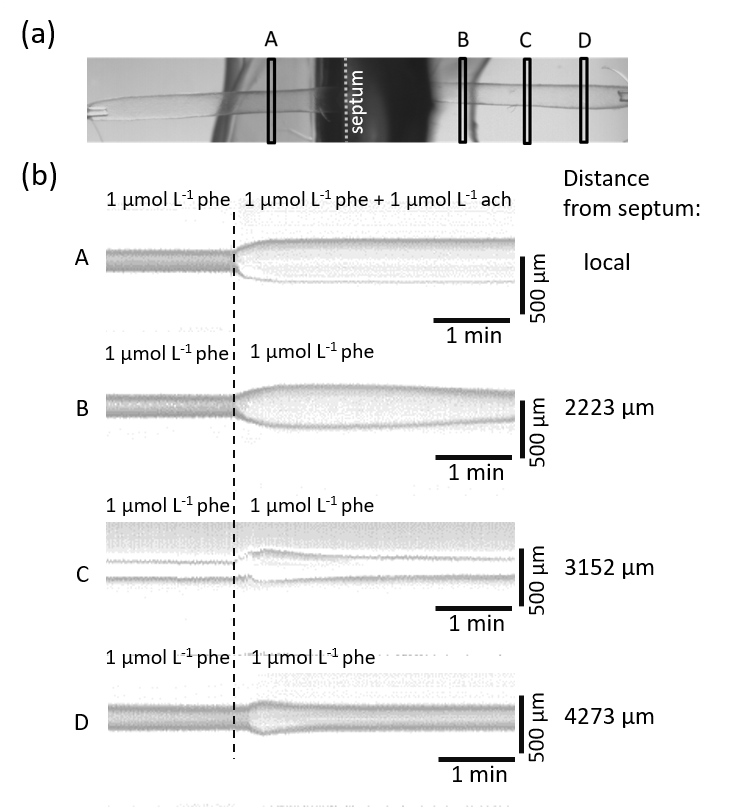

Supplement: Supplementary file 7 [file APHA-224-na-s007.tif]
